# Gibberellin-related genes regulate dwarfing mechanism in wintersweet

**DOI:** 10.3389/fpls.2022.1010896

**Published:** 2022-09-26

**Authors:** Ting Zhu, Bin Liu, Ning Liu, Jie Xu, Xingrong Song, Shuangjiang Li, Shunzhao Sui

**Affiliations:** ^1^Key Laboratory of Horticulture Science for Southern Mountainous Regions of Ministry of Education, Chongqing Engineering Research Center for Floriculture, College of Horticulture and Landscape Architecture, Southwest University, Chongqing, China; ^2^Horticulture Research Institute, Sichuan Academy of Agricultural Sciences, Chengdu, China

**Keywords:** gibberellic, DELLA, *Chimonanthus praecox*, plant height, WGCNA

## Abstract

*Chimonanthus praecox* (wintersweet) is an important cut flower and pot plant with a high ornamental and economic value in China. The development of dwarf wintersweet varieties has become an important research topic for the wintersweet industry. The lack of natural dwarf germplasm has hindered research into the molecular mechanisms of developing dwarf wintersweet, limiting its cultivation. After a long-term investigation and collection of germplasm resources of *C. praecox*, we obtained the germplasm of a dwarf *C. praecox* (dw). Here, the dwarf and normal *C. praecox* (NH) were used to identify the types of hormones regulating dw formation using phenotypic identification and endogenous hormone determination. Differentially expressed genes in the dw and NH groups were screened using transcriptome analysis. The functions of key genes in the dwarf trait were verified by heterologous expression. It was found that the internode length and cell number were significantly reduced in dw than in NH, and the thickness of the xylem and pith was significantly decreased. The dwarfness of dw could be recovered by exogenous gibberellic acid (GA) application, and endogenous GA levels showed that the GA4 content of dw was substantially lower than that of NH. Transcriptome differential gene analysis showed that the elevated expression of the *CpGA2ox* gene in the GA synthesis pathway and that of *CpGAI* gene in the signal transduction pathway might be the key mechanisms leading to dwarfing. Combined with the results of weighted gene co-expression network analysis, we selected the *CpGAI* gene for analysis and functional verification. These results showed that CpGAI is a nuclear transcriptional activator. Overexpression of *CpGAI* in *Populus tomentosa* Carr. showed that *CpGAI* could lead to the dwarfing in poplar. We analyzed the dwarfing mechanism of *C. praecox*, and the results provided a reference for dwarf breeding of wintersweet.

## Introduction

*Chimonanthus praecox* (wintersweet) is a traditional ornamental wood specialty plant in China. Wintersweet has a long history of flower culture with a wide variety of cultivated species and is an excellent rare garden winter flower. It is also a valuable potted and cut flower material with good market value (Lin and Chen, 2020). As potted plants can be moved and are easy to manage, they are suitable for interior decoration in public places, such as restaurants, industries, schools, and hospitals, and meet the needs of family horticulturists. Recently, wintersweet potted plants have been receiving increasing attention from the industry (Xiao, 2021). The plant height of wintersweet is generally approximately 3 m. Pruning and exogenous gibberellin inhibitors, such as chlormequat chloride, paclobutrazol, and daminozide, are used to produce dwarfs of wintersweet (Yuan and Song, 2012; Du et al., 2015). These methods have several disadvantages, namely harmful effects on growth, high labor cost, and difficulty in ensuring stability in the later period. Currently, no good solution exists for controlling the height of wintersweet plants.

Plant architecture is a result of the continuous or periodic division of the meristem. In dicotyledons, plant height is promoted mainly by the stem apical meristem (SAM), whereas the lateral meristem increases the stem diameter (Wang et al., 2018). Studies have also shown that plant height and stem diameter are positively correlated (Rezaee et al., 2009). Plant height exhibits plasticity, which depends on the coordinated regulation of environmental signals and plant hormones, and mainly regulates stem elongation by promoting cell elongation and increasing cell number (Bai et al., 2012; Oh et al., 2014). Gibberellin acid (GA), a tetracyclic diterpenoid plant hormone, is one of the most important hormones regulating plant height. Significant changes in expression of genes related to GA synthesis or signaling pathways, or mutations can lead to changes in plant height. GA biosynthesis is catalyzed by the following key enzymes, including geranylgeranyl pyrophosphate synthase (GGPS), copalyl diphosphate synthase (CPS), kaurene synthase (KS), kaurene oxidase (KO), kaurenoic acid oxidase (KAO), GA20-oxidase (GA20ox), and GA3-oxidase (GA3ox). GA deactivation is catalyzed by GA2-oxidase (GA2ox) (Hedden and Phillips, 2000). In rice, the *OsEATB* gene negatively regulates internode length by inhibiting KS transcription (Qi et al., 2011); overexpression of *OsWOX3A* results in shorter internode length by inhibiting KAO transcription (Cho et al., 2016). Both responses lead to dwarfing caused by the decreased expression of GA synthesis-related genes. A typical case of dwarfing caused by the mutation of the *sd1* gene in rice, which codes for the downstream gene *GA20ox-2* that is involved in GA synthesis, led to the “green revolution” (Ashikari et al., 2002). In GA signal transduction, DELLA protein degradation and inactivation result in internode elongation. When the N-terminal domain of the DELLA protein is mutated, DELLA protein degradation is blocked and it becomes a constitutive repressor; hence, plants show a dwarfing phenotype insensitive to GA (Dill et al., 2001; Fu and Harberd, 2003; Fu et al., 2004). In *Arabidopsis thaliana*, the NAC transcription factor *JUB1* inhibits stem elongation by increasing the transcription level of DELLA, a key factor in the GA signaling pathway (Dill et al., 2001; Shahnejat-Bushehri et al., 2016). Another representative case of the “Green Revolution” (semi-) dwarf dry wheat is that two genes encoding DELLA protein—*Rht-B1* and *Rht-D1*—are insensitive to GA signal, resulting in shorter plant height (Peng et al., 1999).

Most studies on plant height have been conducted in herbaceous model plants, such as rice, wheat, and *Arabidopsis*, while studies on the regulation mechanism of plant height in woody plants are limited. In a dwarfing study on peach, it was found that the mutation of cytosine to thymine in the sequence GID1c led to the conversion of serine (S) to phenylalanine (F). Even in the presence of GA, GID1c*^S^*^191*F*^ cannot interact with the growth inhibitor DELLA1, resulting in GA-insensitive dwarfs (Cheng et al., 2019). Studies on the crape myrtle, an ornamental woody plant, have shown that auxin plays a key role in cell division in the SAM and that auxin and GA4 interact to regulate the internode length (Ju et al., 2018). Most studies on wintersweet are based on characteristics, such as flower senescence and flower bud dormancy (Huang et al., 2019; Li et al., 2020), and the molecular mechanism of dwarfing has not been reported. The first dwarf material identified in this study is ideal for analyzing the dwarfing mechanism. The dwarfing mechanism of *C. praecox* was studied using cell observation, hormone determination, transcriptome analysis, and transgenic verification. This study also provides a reference for analyzing the molecular mechanism of dwarfing characteristics in woody plants.

## Materials and methods

### Phenotype and hormone treatment

This study used dwarf (dw) and common *C. praecox* (NH) as experimental materials. In October 2020, grafting of the two accessions was performed at the teaching base of the College of Horticulture and Landscape Architecture of Southwest University (Chongqing, China). In March 2021, the scions sprouted for follow-up experiments.

Twelve branches of each accession were randomly selected to measure branch length and internode number. Branch length was measured when the scion began to grow on March 04, 2021, and the internode number was counted when internodes became distinct on March 31, 2021. Both measurements and statistics were performed every 7 days until the summer shoot growth was completed. The internode length was obtained by dividing the branch length by the number of internodes. Multiple comparisons and related phenotypic regression analyses were performed using SPSS 20.0.

Six branches with a length of 12 cm and three nodes were selected from dw, and the whole branch was sprayed with GA at a concentration of 150 mg/L. The treatment was administered at 9:00 a.m. on the 1st, 5th, and 10th days. Before treatment, 6 dw and 6 NH plants of the same height and node number were selected and sprayed with water as a control. These branches’ height and the number of internodes were observed 14 days later.

### Histological observation of the stem

The dw and NH branches with four internodes were selected for histological observation, and the stem tip, cross section, and longitudinal section of the stem segment were prepared. One branch was considered as one biological repeat and a total of three biological repeats were considered.

The tender stem tip was dissected using paraffin sections as follows. Fresh tissue from the stem tip was fixed with 50% fixative solution for more than 24 h. Dehydration boxes were placed into the dehydrator and gradient alcohol for dehydration. The wax-soaked tissues were embedded in an embedding machine. Samples were sectioned on a Leica RM2016 slicer with a thickness of 4 μm. After staining with safranin and solid green, the sections were sealed with xylene transparent and neutral gum, respectively; then, they were observed and photographed using a NIKON ECLIPSE E100 optical microscope.

The tissue of the stem segment was hard (the fourth internode from the tip down), and the cross section was obtained by hard-tissue slicing. The cells were fixed with 70% formaldehyde-acetic acid-ethanol fixative (FAA) for 48 h, dehydrated, molded, embedded successively, and then sectioned using a Leica HistoCore AUTOCUT hard tissue slicer at a thickness of 10 μm. Safranin and solid green staining, and the images were observed under a NIKON ECLIPSE E100 optical microscope and photographed using Nikon DS-U3.

The length of the stem tip was measured using the Nano Measurer software (Fudan University), and the tissue thickness and cell size of the stem sections were calculated using CaseViewer software. The number of cells in the longitudinal section of the internode was obtained by dividing the average internode length by the average cell length (Ripetti et al., 2008).

### Determination of endogenous gibberellic acid content

The stem tip and the first two stem segments (excluding leaves) of *C. praecox* branches obtained in early April 2021 were used for endogenous GA content determination in three biological repeats according to the method of Pan et al. (2010). Approximately 0.5 g of fresh sample was ground to a dry powder using liquid nitrogen and placed in a glass test tube. A mixture of isopropanol-water-hydrochloric acid and 8 μL of 1 μg/mL internal standard solution was added to the glass tube and oscillated for 30 min at 4°C. Dichloromethane was then added, and the mixture was oscillated for 30 min at 4°C. The solution was centrifuged at 6,000 *g* for 5 min at 4°C, and the lower organic phase was obtained. The organic phase was dried using nitrogen and redissolved in methanol (0.1% formic acid). After centrifugation, the supernatant was passed through a 0.22-μm filter membrane and analyzed using high-performance liquid chromatography-tandem mass spectrometry (HPLC-MS/MS).

### Transcriptomic and quantitative real-time PCR analysis

According to the statistical results of previous pre-experiments, we roughly divided the growth of wintersweet into three stages. The first stage occurred in mid-early March (T1 group), when wintersweet began to grow; the second stage occurred in mid-April (T2 group), when the growth rate of wintersweet was fast, and the third stage occurred in early May (T3 group), when it began to grow slowly. The stem tip and first two stem segments (excluding leaves) were selected for all three groups, and 10 branches were mixed as one biological replicate, with three biological replicates in each of the three groups.

Total RNA was extracted using the TRIzol kit (Tiangen, Beijing, China); mRNA was purified, and a cDNA library was constructed. Illumina HiSeq 4000 and PE 150 sequencing strategies were used for sequencing. Raw sequencing data were filtered to obtain clean data. The transcript was spliced using Trinity software; redundancy was removed using CD-HIT (v4.8.1), and splicing quality was evaluated using the BUSCO software. Gene expression was calculated using RSEM software, and genes with FDR < 0.05 and | log2 (Fold Change)| > 1 were considered to be differentially expressed genes (DEGs).

To describe the pattern of genetic association among different samples and identify highly synergistic gene sets, we performed a weighted gene co-expression network analysis (WGCNA) using the R package “WGCNA” (Zhang and Horvath, 2005) and visualized using Cytoscape v1.7.251^1^.

To verify the RNA-seq data, the expression patterns of nine related DEGs were analyzed. Specific primers designed using Primer6 were used for qRT-PCR in the Bio-Rad CFX96 system (Supplementary Table 1). Relative gene expression levels were calculated using the 2^–ΔΔ^*^CT^* method (Livak and Schmittgen, 2001). Three biological replicates and three technical replicates were performed.

### Gene cloning and *CpGAI* bioinformatics analysis

The first-strand cDNA was synthesized from RNA according to the manufacturer’s instructions using a PrimeScript RT kit with gDNA Eraser (TaKaRa, Dalian, China). The *CpGID1, CpGAI*, and *CpGID2* genes were amplified using the Pfu DNA polymerase kit (TransGen, Beijing, China) and sequence-specific primers (*CpGID1*-F/R, *CpGID2*-F/R, and *CpGAI*-F/R, respectively) (Supplementary Table 2). The PCR products were cloned into the pMD19-T vector (Takara, Shiga, Japan) for sequencing.

Multiple amino acid sequences were compared using ClustalW, and the results were plotted using JalView. A phylogenetic tree was constructed using MEGA7.0 software based on the neighbor joining method, and bootstrap analysis was performed with 1,000 replications. The amino acid sequences of other plant used in this alignment and phylogenetic trees were obtained from the National Center for Biotechnology Information (NCBI).

### Subcellular localization of *CpGAI*

Specific primers were designed to determine the subcellular localization of *CpGAI* (Supplementary Table 2). The ORF of *CpGAI* without a stop codon was cloned into the N terminus of the pCAMBIA1300 vector *GFP* (green fluorescent protein) gene using a seamless cloning kit (Yugong Biolabs, Jiangsu, China). The fusion recombinant plasmid *pCAMBIA1300-CpGAI* and empty vector *pCAMBIA1300* (positive control) were transformed into *Agrobacterium tumefaciens* and then transfected into onion epidermal cells. Simultaneously, the untransfected onion epidermis was used as the blank control. Onion cells were cultured in MS medium at 28°C in the dark for 24 h, and the nuclei of onion cells were stained with 10 μg/mL 4′, 6-diamidino-2-phenylindole (DAPI). Observations were performed using a Zeiss LSM 800 confocal excitation light microscope.

### Transcriptional self-activation activity of *CpGAI*

Specific primers were designed to determine whether *CpGAI* has transcriptional self-activation activity (Supplementary Table 2). Similarly, *CpGAI* was cloned into the yeast expression vector pGBKT7 (Clontech, Shiga, Japan) using a seamless cloning method to construct the vector *pGBKT7-CpGAI*. According to the instructions of the yeast transformation kit (Coolaber), *pGBKT7-CpGAI*, *pGBKT7* (negative control), and *pGBKT7-TF39* (positive control) were transformed into the yeast strain Y2HGold. Exactly 10 μL of 10^0^, 10^–1^, 10^–2^, and 10^–3^ diluents of yeast cells were selected on SD/-Trp, SD/-Trp, and X-α-gal (5-bromo-4-chloro-3-indoxyl-α-D-galactopyranoside) media. The presence of yeast plaque and whether the plaque turned blue were observed to determine whether the CpGAI protein had transcriptional activation activity. The yeast media described above were purchased from TaKaRa (BioTech, Dalian, China). All yeast cells were cultured at 29°C for 2–3 days.

### *CpGAI* overexpression in *Populus tomentosa* Carr.

As described by Jia et al. (2010), *Agrobacterium*-mediated leaf disc transformation was performed in *P. tomentosa*. Primers for identification and expression analysis of the transgenic-positive seedlings are shown in Supplementary Table 2.

## Results

### Phenotype, exogenous gibberellic acid treatment, and endogenous gibberellic acid content determination of dwarf and NH

**FIGURE 1:**
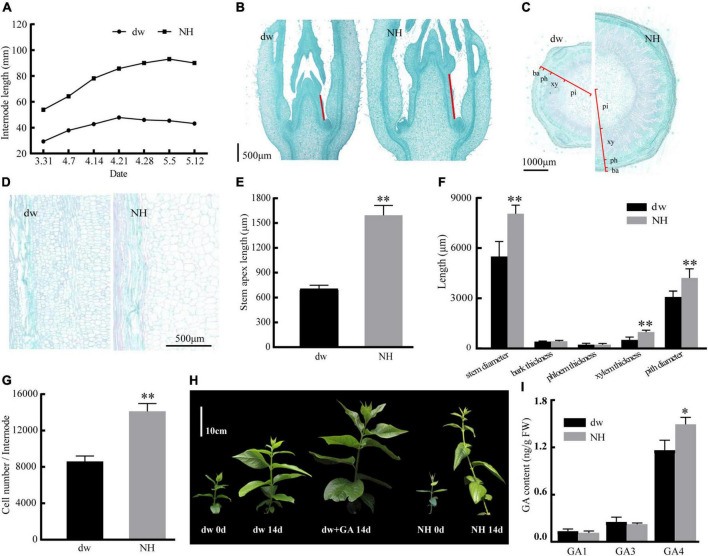
Response of internode length, slicing of dw and NH, dw to exogenous GA treatment. **(A)** Internode length in different periods. Panels **(B,E)** are the paraffin sections of the stem tip, with red lines indicating the length of the stem tip. Panels **(C,F)** are transverse sections of the stem. ba, bark; ph, phloem; xy, xylem; pi, pith. Panels **(D,G)** are the longitudinal sections of the stem. **(H)** The effect of hormone treatment on the growth of dw and NH. **(I)** Endogenous GA content. The * and ** indicate a significant difference from NH at *p* < 0.05 and *p* < 0.01, respectively, as determined by the Student *t*-test.

The statistical results of the internode length of the two accessions of *C. praecox* showed that the internode length of NH was almost three times that of dw (Figure 1A). The average stem tip lengths of NH and dw were 1,562 and 713 μm, respectively, in the paraffin sections (Figures 1B,E), indicating significant differences between the two accessions in the early stage of branch development. The transverse section of the stem (Figures 1C,F) showed that the difference in stem thickness of dw and NH was caused mainly by the difference in xylem thickness and pith diameter. Longitudinal hard-tissue slicing showed that the size of the dw cells was significantly smaller than that of NH cells (Figures 1D,G).

The initial length of dw and NH was 12 cm, and three internodes were marked. After 14 days, it was found to be 36 cm with six internodes for NH (Figure 1H) and 24 cm with five nodes for dw. The average branch length of dw treated with exogenous GA was 34 cm with six nodes, which was 41.7% more than the length of untreated branches, almost the same as NH. Exogenous gibberellin treatment was strongly resilient for the branch length of dw. These results suggested that the decrease in endogenous GA may cause dwarfing. Moreover, the endogenous content of GA4 in NH was determined to be significantly higher than that in dw (Figure 1I), which further confirmed that endogenous GA plays an important role in regulating plant height in *C. praecox*.

### Transcriptome sequencing, differential genes, and functional enrichment analysis

A total of 397.53 Gb of raw reads were obtained. After quality control, 388.24 Gb clean reads were obtained. The contents of N50 and GC were 2,357 bp and 45%, respectively. Genes were annotated using functional databases (Supplementary Figures 1A,C, 2). Principal component analysis showed good biological repeatability among samples (Supplementary Figure 1B). Nine genes were selected to test the transcriptome results’ reliability and consistency. Their expression levels were assessed by qRT-PCR. The expression patterns obtained by qRT-PCR and RNA-seq analyses were similar (Supplementary Figure 3A). Linear regression analysis showed that the goodness of fit *R*^2^ reached 0.92 (Supplementary Figure 3B), indicating the reliability of the transcriptome data.

**FIGURE 2:**
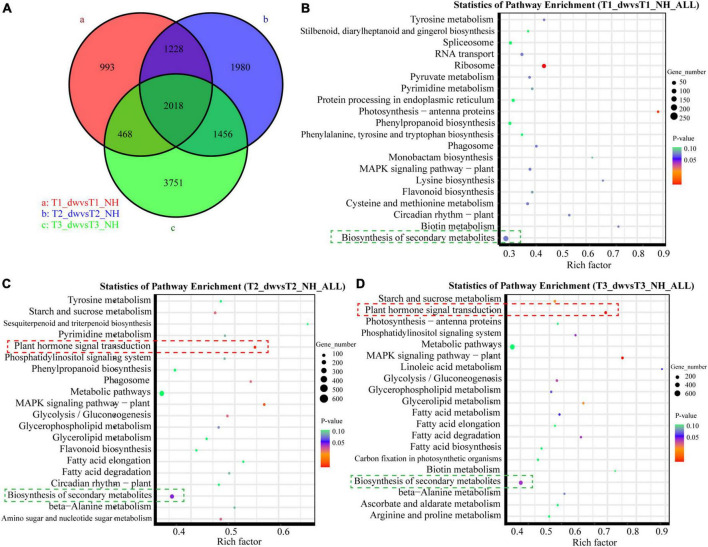
Venn diagram and KEGG function analysis of DEGs in T1, T2, and T3 groups. **(A)** Venn diagram of DEGs in three groups. Panels **(B–D)** are scatter plots of the first 20 KEGG enrichment pathways of DEGs at T1, T2, and T3 stages, respectively.

There were differences among the T1, T2, and T3 groups (Figure 2A). Scatter plots of the first 20 pathways in the three groups were constructed based on the significance of enrichment. A pathway with a *P*-value less than 0.05 was defined as a significantly enriched pathway. In the T2 (Figure 2C) and T3 (Figure 2D) groups, 70 and 89 DEGs belonged to the plant hormone signal transduction pathway (red box), respectively, which was also the most significantly enriched pathway in the two groups. It suggested that plant hormone signal transduction changes greatly during T2 and T3 stages, which is probably an important pathway. Notably, all the first 20 pathways enriched in the T1 (Figure 2B), T2, and T3 groups include biosynthesis of secondary metabolites (green box). In addition, it was significantly enriched in the T2 (*p* = 0.0406) and T3 (*p* = 0.0378) pathways, and many terpenoids genes were found in this pathway.

**FIGURE 3:**
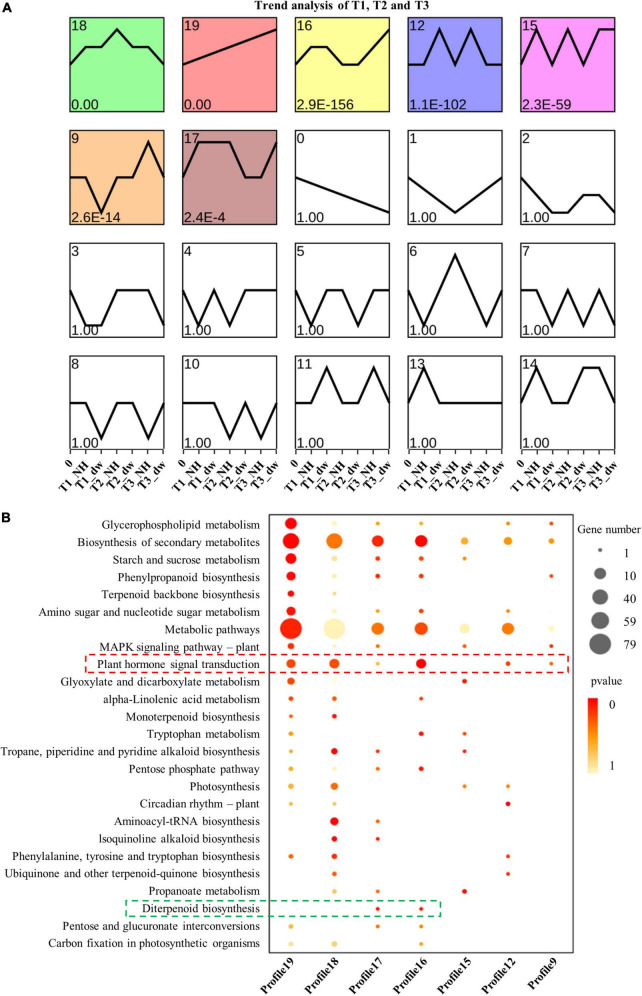
Trend analysis and KEGG enrichment analysis of significant cluster profile. **(A)** Trend cluster analysis of all DEGs at T1, T2, and T3; colored boxes represent profiles with significant differences. **(B)** KEGG enrichment and scatter plots of all genes in trend profile.

The DEGs of dw and NH at T1, T2, and T3 stages were clustered according to the change in expression to determine the up- and down-regulation of all the DEGs (a total of 11,894) in the three stages (Figure 3A). The results showed that profiles 19, 12, and 16 were up-regulated; 18, 9, and 15 were down-regulated, and profile 17 was up- and down-regulated. Furthermore, genes with a significant enrichment trend were analyzed using KEGG enrichment analysis (Figure 3B). Profiles 19, 18, 17, 16, 12, and 9 were enriched in the plant hormone signal transduction pathway (red box), and profiles 16 and 17 had differential genes enriched in the diterpenoid biosynthesis pathway (green box). These profile enrichment analyses indicate that GA synthesis and signal transduction are key pathways. Hence, future studies will be focused on these key pathways.

### Differentially expressed genes involved in gibberellic acid synthesis and signaling pathways

**FIGURE 4:**
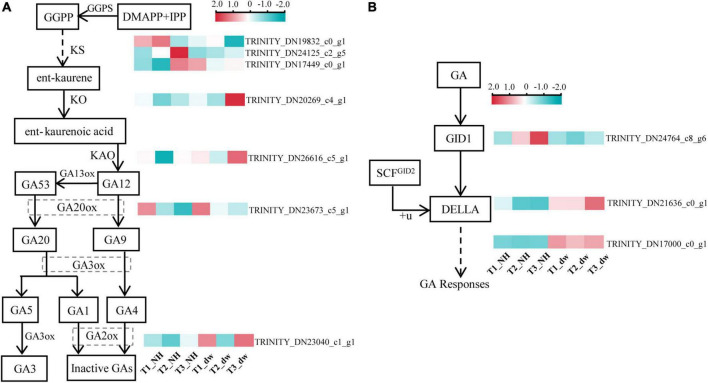
Analysis of DEGs related to GA synthesis and signal pathways. **(A)** DEGs in the GA synthesis pathway. **(B)** DEGs in GA signaling pathway. GGPS, geranylgeranyl pyrophosphate synthase; KO, ent-kaurene oxidase; KAO, ent-kaurenoic acid oxidase; GA20ox, gibberellin 20 oxidase; GA2ox, gibberellin 2-β-dioxygenase; GID1, gibberellin Insensitive Dwarf1; GAI, gibberellin-acid Insensitive; GID2, gibberellin Insensitive Dwarf2.

Gibberellic acid plays an important role in regulating plant height. The above exogenous hormone treatments proved that GA played a significant role in regulating plant height in *C. praecox*. Combined with the results of the KEGG analysis of the transcriptome, we believe that the synthesis and signal transduction of GA may play a crucial role in dwarfness. Therefore, we analyzed the differential transcriptional expression of the GA pathway (at least one of the three periods was different) and drew the following heat map (Figures 4A,B).

Three transcripts (TRINITY_DN19832_c0_g1, TRINITY_DN24125_c2_g5, and TRINITY_DN17449_c0_g1) were found that were related to the GGPS enzyme, which catalyzes the synthesis of the diterpenoid precursor GGPP (Sun and Kamiya, 1994), and the transcriptional level of two of them in NH was significantly higher than that in dw. The expression of KO (TRINITY_DN20269_c4_g1), KAO (TRINITY_DN26616_c5_g1), and GA20ox (TRINITY_DN23673_c5_g1) transcripts was significantly higher in dw than that in NH at T3. GA2ox (TRINITY_DN23040_c1_g1) inactivates bioactive GA1 and GA4 (Hedden and Phillips, 2000), and its expression was significantly upregulated at the T1 and T3 stages. Thus, GA2ox may inactivate endogenous GA in dw. We also analyzed the transcriptional levels of three key genes in the GA signal transduction pathway (Figure 4B), including the GA receptor GID1, F-box protein GID2, and DELLA protein (Sun, 2010), in which DELLA is a negative factor for gibberellin signal transduction (Locascio et al., 2013). One GID1 transcript (TRINITY_DN24764_c8_g6) was downregulated among the DEGs in the T2 and T3 stages. The level of a typical member of the DELLA protein family, GAI1-like (TRINITY_DN21636_c0_g1), which we call *CpGAI*, was significantly higher in dw than that in NH at T2 and T3. Only one transcript of GID2 (TRINITY_DN17000_c0_g1) was upregulated in dw.

### Weighted gene co-expression network analysis

**FIGURE 5:**
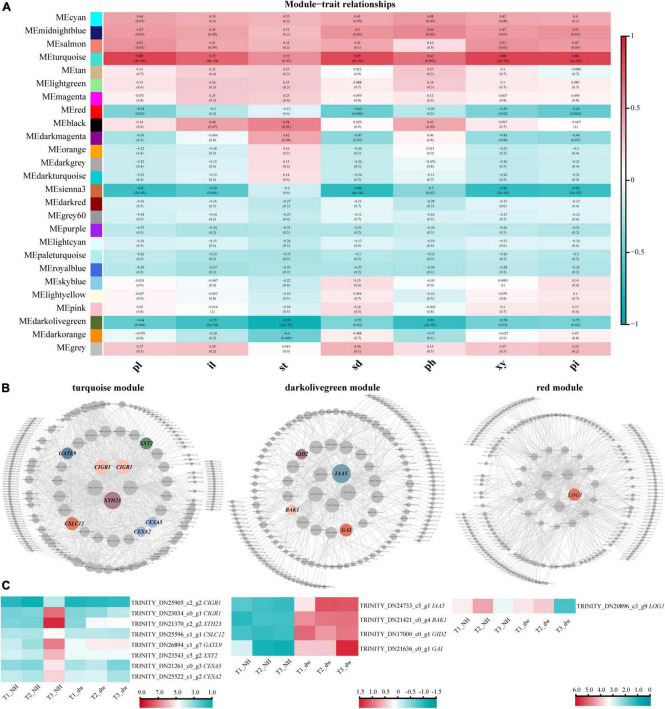
WGCNA and HUB genes of three key modules. **(A)** Association between modules and phenotypic traits. pl, plant height; il, internode length; st, length of stem tip; sd, stem diameter; ph, phloem thickness; xy, xylem thickness; pi, pith diameter. **(B)** HUB gene interaction network. CIGR1, chitin-inducible gibberellin-responsive protein 1; XTH23, probable xyloglucan endotransglucosylase/hydrolase protein 23; CSLC12, probable xyloglucan glycosyltransferase; GATL9, probable galacturonosyltransferase-like 9; XXT2, xyloglucan 6-xylosyltransferase 2; CESA2, cellulose synthase A catalytic subunit 2; CESA5, cellulose synthase A catalytic subunit 5; IAA5, auxin-responsive protein IAA5-like; BAK1, brassinosteroid insensitive 1-associated receptor kinase 1-like; LOG1, cytokinin riboside 5′-monophosphate phosphoribohydrolase LOG1. **(C)** Heat map of HUB gene expression.

To correlate plant height-related genes and phenotypic traits (pl, il, st, sd, ph, xy, and pi), we constructed a module–trait relationship using WGCNA. Among these phenotypes, ph, il, and st had the most direct relationship with plant height. Some studies have shown a strong positive correlation between stem diameter and stem height (Rezaee et al., 2009); therefore, the phenotypic characteristics of sd, ph, xy, and pi are also closely related to plant height. All genes in the 18 samples in WGCNA were divided into 26 modules (Figure 5A). According to *R*^2^ ≥ 0.65 and *p* ≤ 0.005, four modules with the highest correlation with phenotypic characteristics were selected: turquoise, red, sienna3, and darkolivegreen. Genes related to the hormones and cell formation were common in these modules.

Module and trait correlation analysis showed that the modules most related to the phenotypes of pl, il, and st were ‘turquoise’ (positive correlation) and ‘darkolivegreen’ (negative correlation), indicating that the HUB genes of these two modules are likely to play an important role in regulating plant height (Langfelder and Horvath, 2008). The analysis of turquoise (positive correlation) module (Figure 5B, left) revealed eight HUB genes; heat maps were drawn (Figure 5C, left), and two of them were *CIGR1* homologous genes (TRINITY_DN25905_c2_g2, TRINITY_DN23034_c0_g1) of the GRAS protein family. Several genes may be involved in cell wall biosynthesis, including *GATL9* (TRINITY_DN26894_c1_g7), *CESA2* (TRINITY_DN25522_c1_g2), *CESA5* (TRINITY_DN21261 _c0_g3), *XTH23* (TRINITY_DN21370_c2_g2), *CSLC12* (TRINITY_DN25596_c1_g1), and *XXT2* (TRINITY_DN23543 _c5_g2).

In the dark olive green (negative correlation) module, we identified four HUB motifs (Figures 5B,C, middle) related to hormone signal transduction. The genes involved in GA signal transduction were *GID2* (TRINITY_DN17000_c0_g1), *GAI* (TRINITY_DN21636_c0_g1) (GAI was identified again), and the auxin signal transduction gene, *IAA5* (TRINITY_DN24733_c5_g1). A brassinolide signal transduction gene *BAK1* (TRINITY_DN21421_c0_g4) was also identified.

The other two modules, red and sienna3, were analyzed. In the red (negative correlation) module, a hormone-related HUB gene was identified as *LOG1* (TRINITY_DN20896_c3_g9) (Figures 5B,C, right). Although red was a negative correlation module, the expression of *LOG1* in NH was higher than that in dw at T3 stage. No significant difference was observed in the HUB genes in the sienna3 module.

### Gibberellic acid signaling pathway gene cloning and *CpGAI* analysis

The level of active DELLA protein plays a key role in plant height. The above analysis showed that the expression of *CpGAI* in dw was significantly higher than that in NH, which may lead to a higher level of active DELLA protein in dw. To verify whether GID1 and GID2 interact with DELLA protein differently in the GA signal transduction pathway in the two materials, we searched for these genes in the transcriptome database and cloned them in the two materials. A typical DELLA domain was searched in the database, and a *DELLA* homologous gene, *CpGAI* (TRINITY_DN21636_c0_g1), was identified. We also searched for *CpGID1* (TRINITY_DN24764_c8_g6) and *CpGID2* (TRINITY_DN17000_c0_g1) genes. The cloning results showed no difference in the coding region sequences of the three genes in the two accessions (Gene bank accession number: *CpGAI*, OP222008; *CpGID1*, OP222009; *CpGID2*, OP222010). This indicates that the dwarfness of dw was not due to an interaction with the DELLA protein caused by a deletion or mutation of the gene sequence in the GA signal transduction pathway, but by the change at the transcriptional level.

**FIGURE 6:**
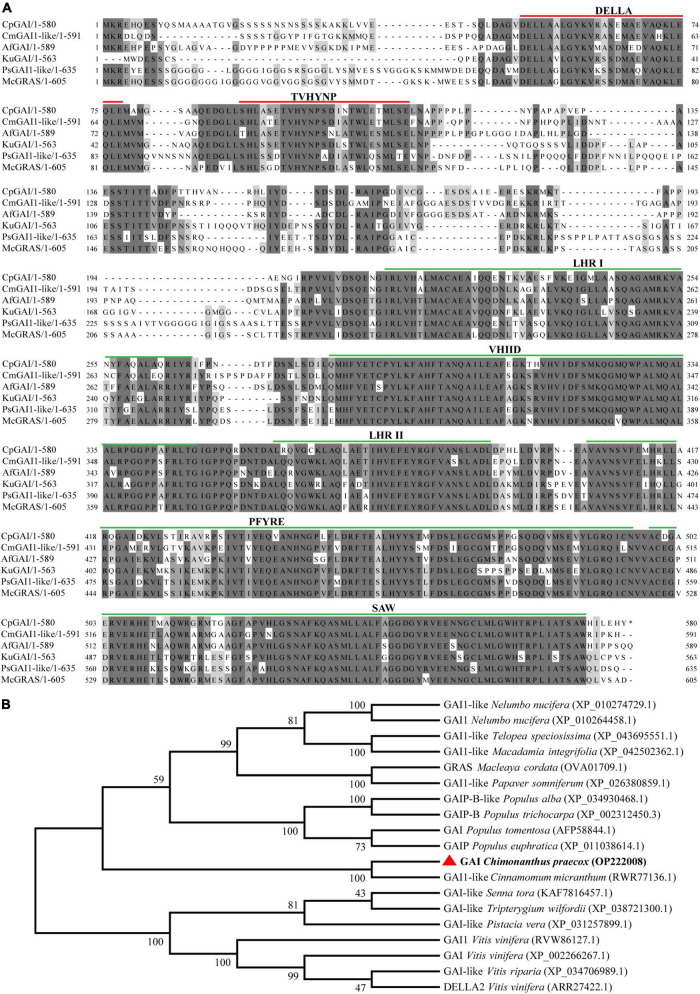
CpGAI amino acid sequence alignment and phylogenetic analysis. **(A)** Multiple sequence alignment of CpGAI protein with the proteins of *Cinnamomum micranthum*, *Aristolochia fimbriata*, *Kingdonia uniflora*, *Papaver somniferum*, and *Macleaya cordata.* The line above shows the location of the conservative area. **(B)** Phylogenetic analysis of the CpGAI protein and GAI of other species; red triangle represents CpGAI.

According to the above research results, we believe that the change in *CpGAI* gene transcription level is one of the key changes that lead to the dwarfing trait in dw; therefore, *CpGAI* was evaluated further. We cloned the full-length *CpGAI* ORF of 1,740 bp in the *C. praecox* cDNA. The amino acid sequence of CpGAI was searched using NCBI pBLAST and compared with homologous proteins of other species (Figure 6A). The conserved domains of CpGAI and CmGAI, AfGAI, KuGAI, PsGAI, and McGRAS have high homology, suggesting that CpGAI might have the functional characteristics of the DELLA protein family. There are typical DELLA domains at the N-terminus of CpGAI, including DELLA and TVHYNP motifs (red lines), which are necessary for sensing GA signals. CpGAI also has typical GRAS domains at the C-terminus, including leucine heptad repeat I (LHR I), VHIID motif, leucine heptad repeat II (LHR II), PFYRE motif, and SAW motif (green lines) (Peng et al., 1997; Pysh et al., 1999). Phylogenetic analysis showed that CpGAI (red triangle) and GAI1-like of *Cinnamomum micranthum* had the highest homology and clustered together (Figure 6B).

**FIGURE 7:**
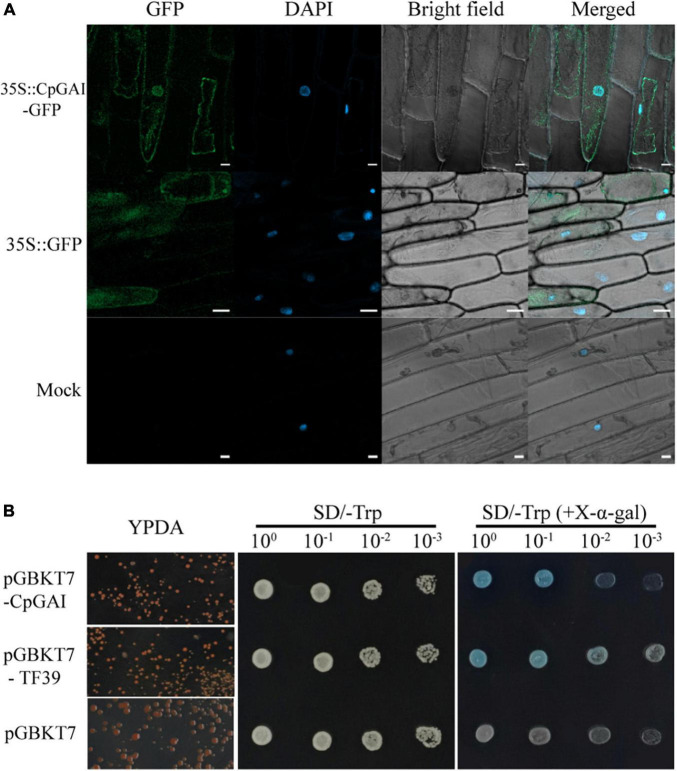
CpGAI is mainly located in the nucleus and has transcriptional self-activation activity. **(A)** Subcellular localization of *CpGAI-GFP* in onion epidermis. The short white line at the lower right of *35S:CpGAI-GFP*, *35S: GFP*, and mock represents 20, 50, and 20 μm, respectively. **(B)** Transcriptional self-activating activity of CpGAI protein in yeast cells. SD/-Trp, medium without tryptophan; SD/- trp (+*X*-α-gal), medium without tryptophan containing 5-bromo-4-chloro-3-indoxyl-α-D-galactopyranoside. pGBKT7-TF39 is the positive control, and pGBKT7 is the negative control.

The GAI is a transcriptional regulator (Fleck and Harberd, 2002). Therefore, we speculated that *CpGAI* might be located in the nucleus. After *A. tumefaciens*-mediated transformation and transient expression, laser confocal observation showed that the *35S:CpGAI-GFP* fusion protein was located mainly in the nucleus with a small amount in the cytoplasm, while GFP in the positive control group containing the empty vector *35S:GFP* was distributed throughout the whole cell, and only blue fluorescence was observed in the mock group, but not GFP (Figure 7A).

We used a yeast system to analyze the self-activating transcriptional activity of CpGAI protein to verify that *CpGAI* can activate transcription. The results showed that *pGBKT7-CpGAI*, *pGBKT7*-*TF39*, and *pGBKT7* strains grew normally on the YPDA medium (Figure 7B). A single colony was picked, shaken, and dropped on SD/-Trp and SD/-Trp (+*X*-α-gal) media. All the strains grew but did not show color on SD/-Trp medium, indicating that the above plasmids were successfully transferred into the Y2HGold strain. The negative control grew on SD/-Trp (+*X*-α-gal) medium but did not show color; the strain transfected with *pGBKT7*-*TF39* and *pGBKT7-CpGAI* recombinant plasmids grew, and the reaction substrate *X*-α-gal turned blue. These results suggest that CpGAI has self-activating transcriptional activity and may play a role in transcription in *C. praecox*.

### Transgenic poplars overexpressing *CpGAI* became shorter

**FIGURE 8:**
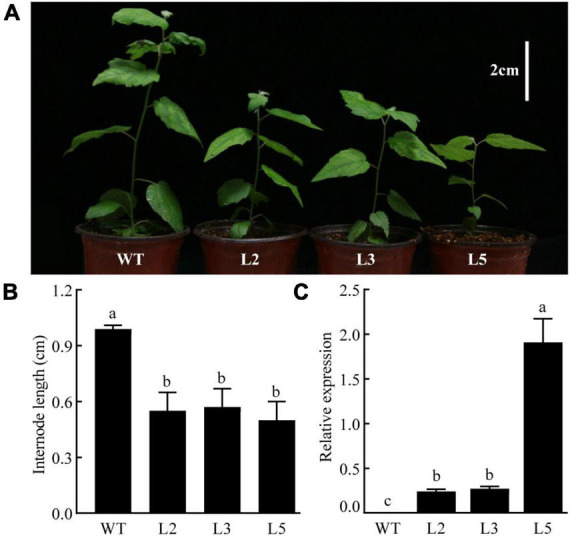
Overexpressed CpGAI lines show reduced plant height (white vertical line in the upper right corner indicates 2 cm). **(A)** Phenotypes of transgenic lines L2, L3, and L5. **(B)** Internode length of transgenic lines and WT. **(C)** Relative expression of *CpGAI* in transgenic lines and WT. Different lowercase letters **(a–c)** above bars indicate significant differences (*p* < 0.05).

The *CpGAI* gene was overexpressed in poplar under the control of the cauliflower mosaic virus 35S promoter to assess the biological function of *CpGAI.* The transgenic lines were identified using PCR, and the expression level of *CpGAI* was determined using qRT-PCR. The results showed that the plant height of the three overexpressing lines (L2, L3, and L5) was lesser than that of the WT (Figure 8A), and the internode length was significantly shorter (Figure 8B). qRT-PCR results showed no expression of *CpGAI* in the WT lines, but it was highly expressed in the three transgenic lines (Figure 8C). Therefore, it is speculated that the increased expression of *CpGAI* may cause dwarfism in dw.

## Discussion

### The lack of endogenous gibberellic acid may be one of the reasons for the dwarfing of dwarf

The height of ornamental plants is very important for enriching horticultural materials. In this study, identifying a dwarf accession of *C. praecox* provided the experimental material for analyzing the mechanism of dwarfing in *C. praecox*. Phenotypic analysis of the dw and NH accessions revealed that decreased internode number and internode length resulted in dwarfing in dw. There was a significant difference in stem tip length between the two accessions in the paraffin sections, which may indicate that internode shortening of dw occurred at the early stage. The transverse section of the stem showed that the thickness of the xylem and pith of dw was significantly smaller than that of NH, which led to a significant difference in the stem diameter. Jiao et al. (2019) overexpressed the *MYB189* gene in poplar and found that the xylem thickness of the transgenic lines was significantly reduced compared to the WT. It was accompanied by a significant decrease in plant height, suggesting that there may be a connection between xylem thickness and plant height.

The GA is an important hormone that controls plant height, mainly by increasing the number and length of cells to promote growth (Richards et al., 2001). Longitudinal sections showed that the number and size of the cells led to a shortening of the dw internodes, which suggests that the dwarfing of dw may be related to the GA pathway. The height of dw can be restored by exogenous GA treatment. Active GA1 and GA4 have been confirmed to play important roles in dwarfing in many plants (MacMillan, 2001). Endogenous GA content analysis showed that GA4 was significantly higher in NH than in dw, which further supports the hypothesis that GA causes dw dwarfing.

### Key differentially expressed genes of gibberellic acid synthesis and signal transduction pathways

The results of KEGG analysis of the transcriptome showed that GA was indeed an important hormone involved in dwarfing in dw, and the transcript expression levels of GA synthesis and signal transduction-related genes in the two materials were analyzed. GA is a diterpene compound that synthesizes the diterpenoid precursor GGPP (Sun and Kamiya, 1994). Tata et al. (2016) transferred the sunflower gene *HaGGPS* into tobacco and found that transgenic tobacco with heterologous expression of *HaGGPS* was taller and had a higher endogenous GA content than the WT plants, indicating that GGPS can increase the content of GA in plants. Our results showed that the expression level of the two *CpGGPS* genes in NH was significantly higher than that in dw, which indicates that NH may synthesize more endogenous GA. The expression of the GA synthesis pathway genes *CpKO, CpKAO*, and *CpGA20ox* in dw was significantly higher than that in NH, which is contrary to our inference; however, the expression of *GA2ox*, which catalyzes the conversion of active GA1 and GA4 into inactive GA (Hedden and Phillips, 2000), in dw was significantly higher than that in NH. Moreover, endogenous GA4 content was substantially lower in dw, which indicates that the content of active GA4 may be decreased because of the high expression of *CpGA2ox*, resulting in dwarfing. In a study on peas, the increase in *GA2ox* expression inhibited the accumulation of active GA (Weller et al., 2009), which is consistent with our presumption of the decrease in endogenous GA4 in dw.

In the signal transduction stage, GID1, DELLA, and GID2 proteins are the three key regulatory factors in GA signal transduction (Sun, 2010). GID1 is the sensory receptor of GA. In *A. thaliana*, the three types of GID1 (a-, b-, and c-) have a higher binding ability to GA4 than the other GAs (Nakajima et al., 2006). The active GA4 content was higher in NH than in dw, which may be the reason for the increased expression of *CpGID1* in NH. The increased expression of *CpGAI* in dw may lead to the accumulation of active DELLA proteins and inhibition of growth in dw.

### HUB genes related to dwarf dwarfing are involved in hormone and cell wall synthesis

Plant height regulation is complex, and we used WGCNA to identify genes closely related to plant height in the entire network. The HUB genes were related mainly to hormones and cell wall formation. The HUB genes related to GA signal transduction pathway included *CpGAI* and *CpGID2*. Two *CIGR1* homologous genes were highly expressed in the NH, similar to plant height studies in rice. The expression of CIGR in all tissues of tall rice plants was higher than that in short rice plants, especially in the young leaf sheaths that contain elongating tissue (Kovi et al., 2011). *IAA5*, an important gene in the auxin signal transduction pathway, was highly expressed in dw. AUX/IAA is an important repressor in the IAA signal transduction pathway, and its related genes regulate plant height. Transgenic rice plants overexpressing *OsIAA1* showed a reduction in plant height (Song et al., 2009). The HUB gene *CpBAK1*, related to brassinolide signal transduction, was highly expressed in dw. *AtBAK1* gene was heterogeneously expressed in rice plants, and the transgenic rice plants showed a semi-dwarfing phenotype during the growth and development stages (Wang et al., 2007). Subsequently, Li et al. (2009) overexpressed a truncated intracellular domain of *OsBAK1* in rice, which led to a dwarfing phenotype, and showed that BAK1 caused dwarfing by affecting plant brassinosteroid signal transduction. The cytokinin-related gene *CpLOG1* was identified as a HUB gene. The *LONELYGUY* (*LOG*) gene has been shown to be necessary to maintain the activity of meristems in rice. It is a cytokinin-activating enzyme that plays a role in the final step of the synthesis of active cytokinins, and its loss of function leads to early termination of the shoot meristem (Kurakawa et al., 2007). Previous phenotype results showed that the number of stem segments in dw was less than that in NH, suggesting that the low expression of *CpLOG1* in dw might lead to the early completion of SAM differentiation. Plant hormones interact with one or more other hormones to function (Santner et al., 2009). Therefore, among the identified HUB genes, it is likely that plant height is regulated by the interaction between hormones, and the mechanism of mutual regulation between hormones needs to be further studied.

The identified HUB genes included *CpXTH23, CpCSLC12, CpGATL9* (*GAUT-Like 9*), *CpXXT2*, and *CpCESA2/5*. These genes and their homologous genes are involved in cell wall formation in other plants and influence stem diameter and elongation (Turner and Somerville, 1997; Burn et al., 2002; Cocuron et al., 2007; Kong et al., 2011; Guo et al., 2020; Sowinski et al., 2022). In rice, GA can upregulate the expression of the *OsXTH8* gene, while other hormones have little effect on *OsXTH8* (Jan et al., 2004). The expression of *XTH23* in *A. thaliana* was significantly upregulated by GA treatment (Yokoyama and Nishitani, 2001). The transcriptional level of the *GAUT* gene may be regulated by hormones other than GA (Fan et al., 2021). In crape myrtle, the number of pith cells and xylem cells was significantly higher in exogenous GA4 treated plants than that in the control (Ju et al., 2018). These results demonstrate that GA can regulate cell growth by inducing the expression of genes related to cell wall synthesis.

### Up-regulation of *CpGAI* may lead to dwarfing of dwarf

Mutation in the genes related to GA signal transduction pathway leads to a change in plant height. In peach dwarfing, the GID1 mutation inhibits the interaction with DELLA protein, resulting in DELLA protein accumulation (Cheng et al., 2019). It has also been shown that DELLA protein accumulation can be induced when the DELLA or VHYNP motifs are mutated (Willige et al., 2007). We cloned *CpGAI, CpGID1*, and *CpGID2* to determine whether dwarfing was caused by gene mutations in the GA signal transduction pathway. We found that these three sequences were identical in the two accessions, respectively, which ruled out the dwarfing of dw due to a gene mutation. It was probably due to the significant upregulation of *CpGAI*. It has been confirmed that high-level transcription of the DELLA gene can inhibit stem elongation (Dill et al., 2001; Shahnejat-Bushehri et al., 2016).

Our domain analysis of CpGAI showed that its N-terminal contains a typical DELLA domain, and the C-terminal has a regular GRAS domain. Subcellular localization indicated that CpGAI was located mainly in the nucleus, with a small amount in the cytoplasm. In *A. thaliana*, sugarcane, and other plants, GAI is located in the nucleus (Dill et al., 2001; Chai et al., 2022). The presence of small amount of GFP signals in the cytoplasm might indicate the localization of free GFP that has been cleaved from the fusion protein (GAI-GFP) in the transfected cells (Quattrocchio et al., 2013). Transcriptional activation experiments further demonstrated that *CpGAI* is a typical transcription factor. DELLA represses plant growth and inhibits GA signaling in two ways (Ariizumi et al., 2008): first, the formation of the GA-GID1-DELLA protein complex enhances the interaction between DELLA and SCF^SLY1/GID2^, resulting in the ubiquitination of DELLA proteins by SLY1/GID2 and degradation by the 26S proteasome (Gomi et al., 2004); second, GA binds to GID1 and directly interacts with the DELLA domain to block the inhibitory activity of DELLA and inactivate the DELLA protein, which is independent of the ubiquitin degradation of DELLA protein by GID2 (Ariizumi et al., 2008). In our results, endogenous GA4 content and the expression of *CpGID1* were high in NH, which may enable the GA-GID1 complex to interact with more DELLA domains of CpGAI, thereby inhibiting DELLA activity. At the same time, the high expression of *CpGAI* in dw may lead to the accumulation of active DELLA proteins and inhibit the growth of dw. Therefore, there may be two inhibitory pathways for the DELLA protein in *C. praecox*. We verified the overexpression of *CpGAI* in *P. tomentosa*, a woody plant. The results showed that the overexpressed population’s plant height and internode length decreased significantly, which confirmed the dwarfing function of *CpGAI*. In a recent study on *A. thaliana*, HB40 directly upregulated the expression of *GA2oxs* to reduce bioactive GA, whereas lower levels of active GA led to an increase in DELLA protein levels and inhibited various growth and development processes, including plant height (Dong et al., 2022), which is similar to our results.

**FIGURE 9:**
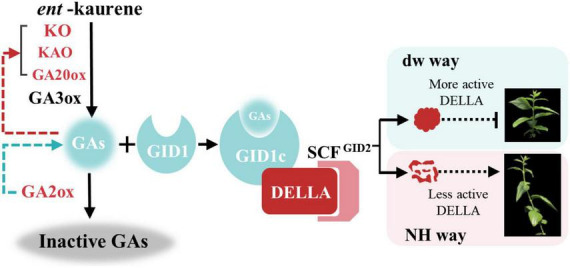
Model of plant height regulation in *C. praecox* (dw and NH). In the synthesis pathway, the red character indicates the up-regulated gene in dw. The red arrow indicates that decreased GA content promotes the up-regulation of synthetic gene expression, and the green arrow indicates that GA2ox reduces the active GA. In the signal transduction pathway, short vertical lines represent more active DELLA proteins that inhibit the elongation of *C. praecox* branches, while dotted arrows represent less active DELLA proteins that allow normal *C. praecox* growth.

Finally, we propose a regulatory mechanism model for dw dwarfing (Figure 9). In the normal plant *C. praecox* (NH), the GA synthesis pathway gene is normally expressed; active GA1, GA3, and GA4 are synthesized, and signal transduction is completed. Finally, DELLA protein is degraded or inactivated by ubiquitin, and wintersweet grows normally. In dw, the KO, KAO, and GA20ox genes that synthesize GA are highly expressed, but GA2ox, which inactivates GA, is also highly expressed and significantly reduces the active GA4 content. The decrease in GA4 results in the increase in the transcription of genes in the synthesis and metabolic pathways and induces dwarfing of dw to some extent. After entering the signal transduction stage, the elevated expression of *CpGAI* in dw leads to increased levels of active DELLA protein. A small amount of GA-GID1 complex is not enough to bind to the functional DELLA protein to inactivate it or be subsequently ubiquitinated, and the active DELLA protein further leads to dw dwarfing.

## Data availability statement

Accession to RNA-seq data: all RNA-sequencing data were deposited in National Center for Biotechnology Information (NCBI) under BioProject accession number: PRJNA869201 (https://www.ncbi.nlm.nih.gov/sra/PRJNA869201). Gene bank accession numbers: CpGAI: OP222008; CpGID1: OP222009; and CpGID2: OP222010.

## Author contributions

SS and SL collected the accessions. TZ and SS designed the research. TZ analyzed the sequencing data and wrote the manuscript. NL performed qPCR. BL, JX, and XS performed graphic drawing. BL and SL edited the manuscript. All authors contributed to the article and approved the submitted version.

## Footnotes

1
https://cytoscape.org/index.html
