# Supplementary material for: Gibberellin-related genes regulate dwarfing mechanism in wintersweet
Source: Front Plant Sci. 2022 Sep 26;13:1010896. doi: 10.3389/fpls.2022.1010896 (PMC9549245; doi:10.3389/fpls.2022.1010896)
Supplement: Supplementary file 1 [file Data_Sheet_1.docx]

**Supplementary Table 1 |** Primers designed for qRT-PCR assay.

| **Unigene ID** | **Unigene name** | **Primer Sequence (5′-3′)** |
| --- | --- | --- |
|  | q*CpActin*-F | GTTATGGTTGGGATGGGACAGAAAG |
|  | q*CpActin*-R | GGGCTTCAGTAAGGAAACAGGA |
|  | q*CpTublin*-F | TAGTGACAAGACAGTAGGTGGAGGT |
|  | q*CpTublin*-R | GTAGGTTCCAGTCCTCACTTCATC |
| TRINITY_DN23543_c5_g2 | q*CpXXT2*-F | ACGAGATCCGCCTCCATCTAACC |
|  | q*CpXXT2*-R | TCTTCTGTGACTACCACCACCTGTT |
| TRINITY_DN21370_c2_g2 | q*CpXTH23*-F | ACTTCCACACCTACTCCATCCTCTG |
|  | q*CpXTH23*-R | TTCTCGGCGTTCTTGAATTGTCTGA |
| TRINITY_DN20269_c4_g1 | q*CpKO*-F | ACGAGGAAACTCTCCAAGGC |
|  | q*CpKO*-R | TCAGCTCAGTGTGGAAGTGG |
| TRINITY_DN26616_c5_g1 | q*CpKAO*-F | ATCCCAGGAACAGCATACCATCA |
|  | q*CpKAO*-R | TCCACGACCTCCATTTCACTCAT |
| TRINITY_DN23673_c5_g1 | q*CpGA20ox*-F | CTCCTTCTCCACCTTCTCTTCTCC |
|  | q*CpGA20ox*-R | ACACCAGTTGCTTCTCCTCCTTC |
| TRINITY_DN23040_c1_g1 | q*CpGA2ox*-F | TGAGAAGGAGACGGCAGGACAC |
|  | q*CpGA2ox*-R | TCAGTCACAGCAGAGCGAAAGAAA |
| TRINITY_DN24835_c2_g3 | q*CpCXE20*-F | CATTGCCTACCACACCGCTCTC |
|  | q*CpCXE20*-R | TCCTTCTTCTGAACACCGCCGAA |
| TRINITY_DN17000_c0_g1 | q*CpGID2*-F | CCTGAATGCGGACGGTGATGAT |
|  | q*CpGID2*-R | CGCTCATCTTGGGCAGTCTTGT |
| TRINITY_DN23034_c0_g1 | q*CpCIGR1*-F | TGCGATGCCGAGAACCTGATAG |
|  | q*CpCIGR1*-R | GCCTTGCTGCCAATGCTTGT |

**Supplementary Table 2 |** Primers for cloning, vector construction, and transgenic seedling identification.

| Purpose of the primers | Primer name | Primer sequence (5′−3′) |
| --- | --- | --- |
| cloning ORF of CpGID1 | *CpGID1*-F | ACCGATTTTCAATGGCTGGG |
|  | *CpGID1*-R | ATACTGCACACCTTCACTCA |
| cloning ORF of CpGAI | *CpGAI*-F | TTTCGAGACAAGGGTAGGTGG |
|  | *CpGAI*-R | GGTGGTTGTTCTGAGCTGAATG |
| cloning ORF of CpGID2 | *CpGID2*-F | CTCCTCTCAATTTTAACAAGCAC |
|  | *CpGID2*-R | TTCAAACCCAGAGCCATCTCT |
| inserting *CpGAI* into vector of pCAMBIA1300 | *CpGAI*-KpnI-F | AACACGGGGGACGAGCTCGGTACCATGAAGAGAGAACACCAAGAGA |
|  | *CpGAI*-XbaI-R | CTTGCTCACCATGTCGACTCTAGAATAGTGTTCGAGAATATGCCAAG |
| inserting *CpGAI* into vector of pGBKT7 | *CpGAI*-BamHI -F | TGGAGGCCGAATTCCCGGGGATCCATGAAGAGAGAACACCAAGAGAG |
|  | *CpGAI*-PstI-R | ATGCTAGTTATGCGGCCGCTGCAGATAGTGTTCGAGAATATGCCAAG |
| identification of transgenic poplar | *CpGAI*-F | CTTGAGGGGTGCGGAATGTCC |
|  | *CpGFP*-R | GGTGGTGCAGATGAACTTCAG |
| expression level of *CpGAI* in poplar | q*CpGAI*-F | AGCAATAGCAGCAGCAGCAAGG |
|  | q*CpGAI*-R | CGAGCCAGGTGTTGATGTCAGAG |

Note: Underlined sequences indicate cleavage sites of the restriction enzyme.

**
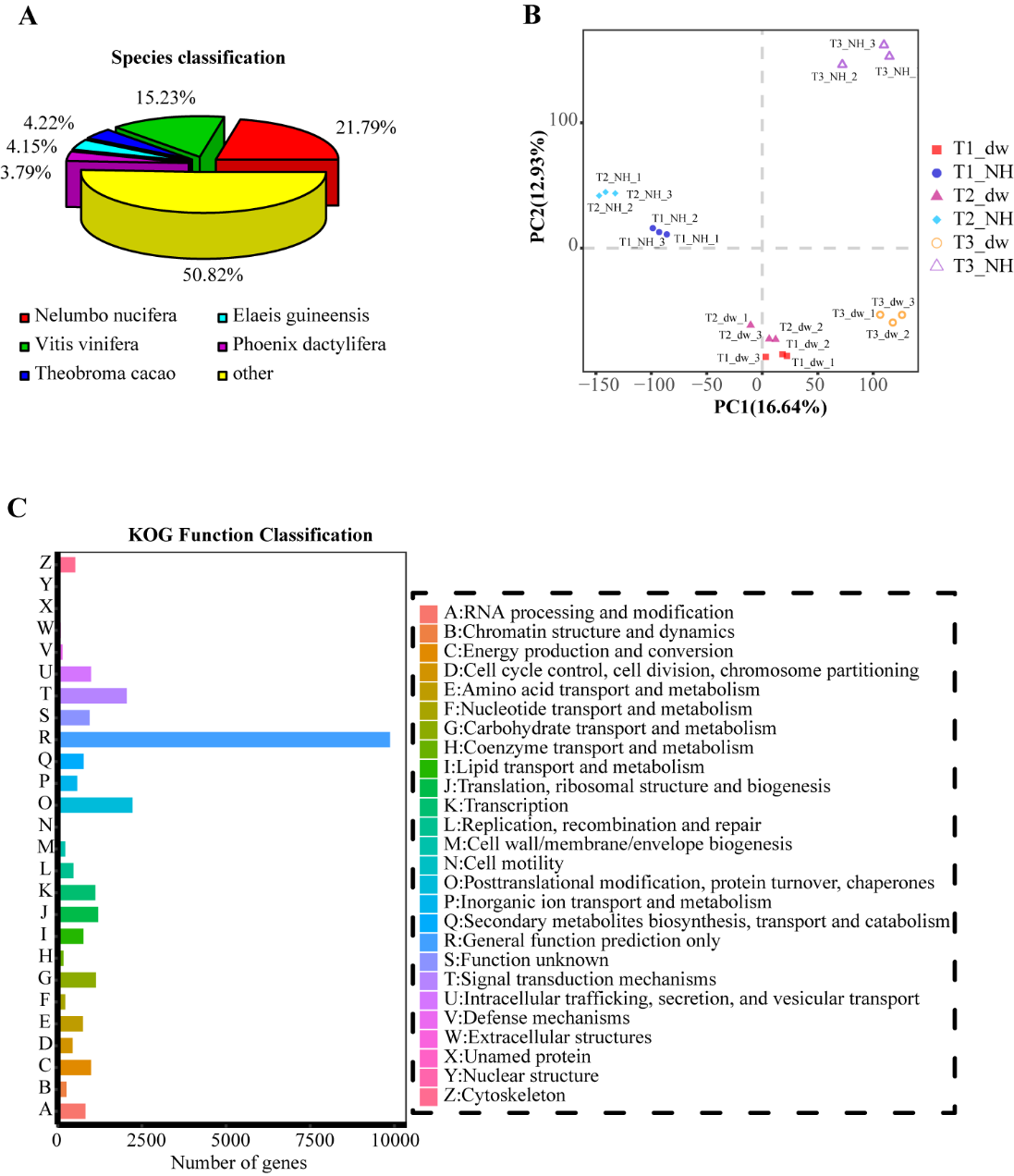
Supplementary Figure 1 |** Annotation ratio of different species **(A)**, principal component analysis **(B)**, and KOG annotation analysis **(C)**.

**
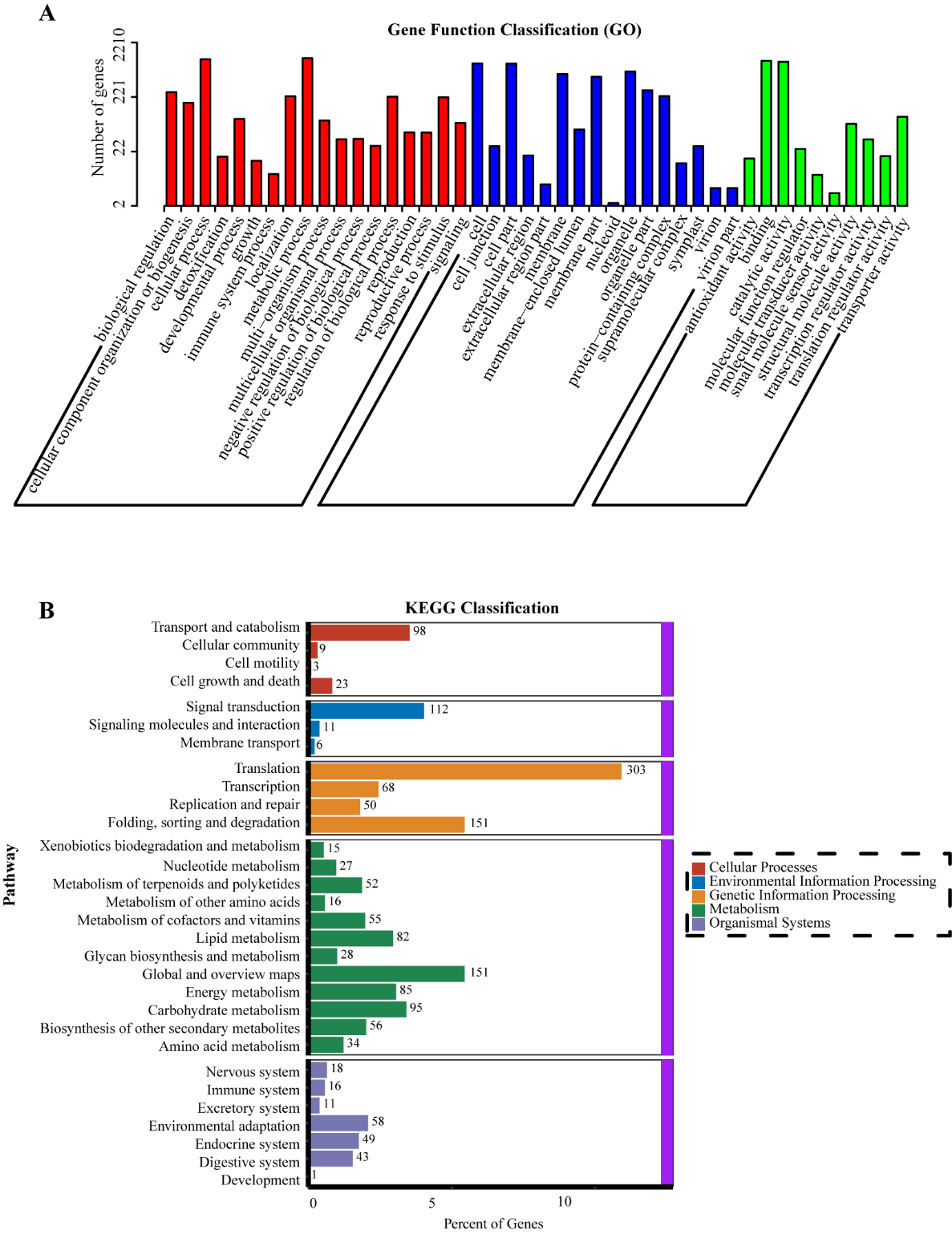
**

**Supplementary Figure 2 |** Gene ontology **(A)** and KEGG **(B)** annotation analysis.

**
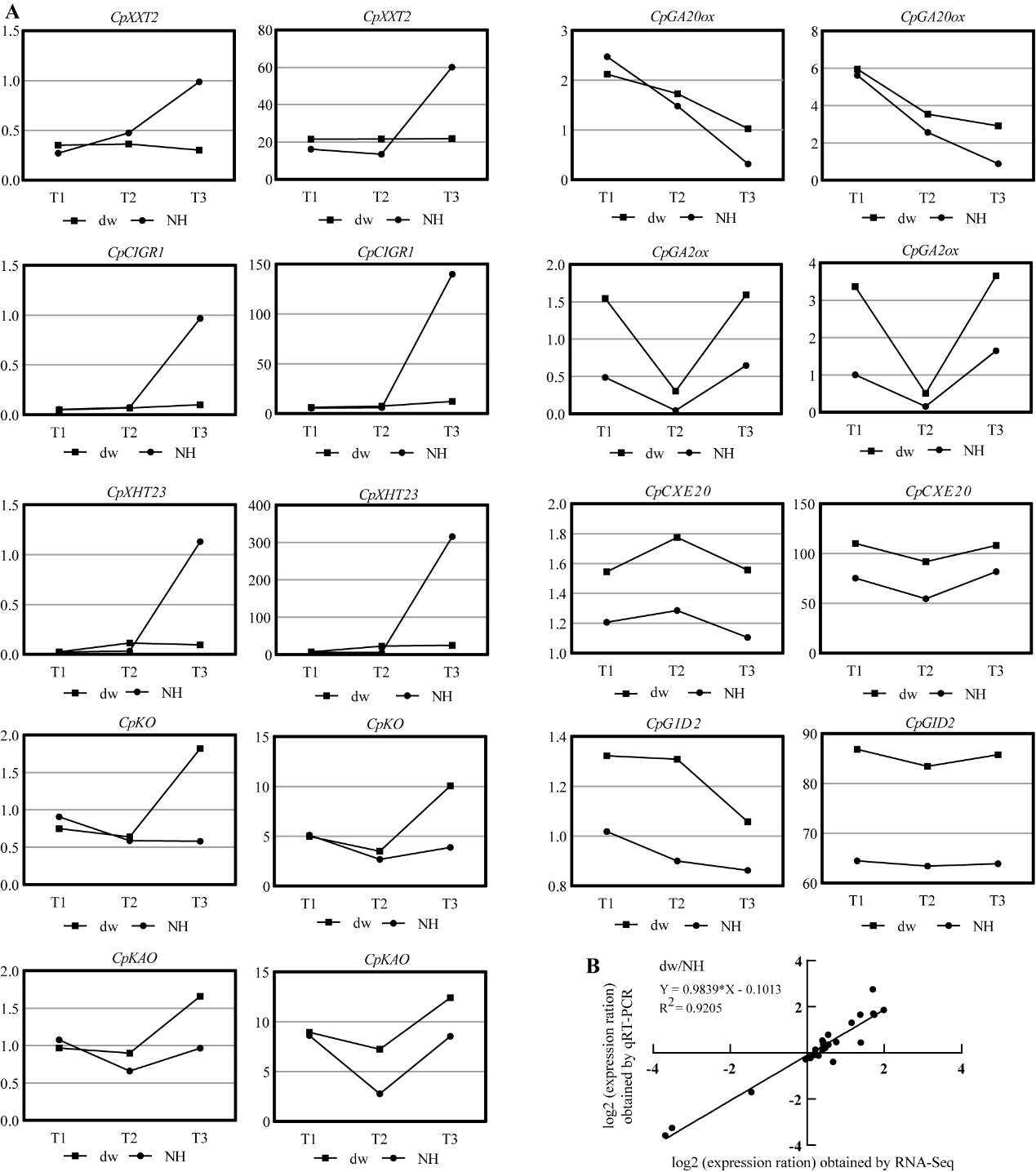
Supplementary Figure3 |** qRT-PCR and transcriptome consistency verification and linear regression analysis of nine related differential genes. **(A)** qRT-PCR results on the left and transcriptome results on the right. T1, T2, and T3 indicate the stages of initial growth, rapid growth, and slow growth, respectively. **(B)** Linear regression analysis of dw and NH.
